# Automated detection of sleep spindles in the scalp EEG and estimation of their intracranial current sources: comments on techniques and on related experimental and clinical studies

**DOI:** 10.3389/fnhum.2014.00998

**Published:** 2014-12-10

**Authors:** Periklis Y. Ktonas, Errikos-Chaim Ventouras

**Affiliations:** ^1^Sleep Study Unit, 1st Psychiatric Clinic, Eginition Hospital, University of Athens Medical SchoolAthens, Greece; ^2^Department of Biomedical Engineering, Technological Educational Institution of AthensAthens, Greece

**Keywords:** sleep spindle, automated detection, inverse problem, intracranial sources, experimental studies, clinical studies

## Introduction

Sleep spindles are short bursts of sleep EEG activity in the range of 11–15 Hz, reflecting central nervous system integrity and considered to promote sleep continuity, learning and memory consolidation processes. This contribution comments on the automated detection of sleep spindles and their intracranial sources, as well as on experimental and clinical studies for the characterization of spindles and their sources, and the study of their functional significance. Supporting literature is provided wherever appropriate, although comprehensive review is out of the scope of this opinion paper.

## Automated detection of sleep spindles

### Detection methods

Visual EEG analysis heuristics, such as counting the number of peaks of the EEG signal within a time window, or counting the number of successive EEG waves having a specific amplitude and period within that window, can be utilized for spindle detection, provided relatively high sampling rates beyond the Nyquist criterion are chosen, e.g., 250 Hz (Principe and Smith, 1986; Ktonas, 1996). However, appropriate EEG pre-filtering with wideband (low-Q) bandpass filters (Shirakawa et al., 1987) may be necessary. Techniques which are based on human pattern recognition can present problems because there is no explicit definition for a sleep spindle. Spindle morphology may vary between the so-called “fast” and “slow” spindles, across subjects, with age and health condition (Nicolas et al., 2001; Ktonas et al., 2009). Appropriate initialization procedures in the detection system, such as adaptively adjusting amplitude or frequency parameters per subject, may help (Ray et al., 2010). Expert system-based approaches, incorporating complex domain knowledge, might be able to address these problems.

Spindle detection can be based on spectral analysis implemented via the Fast Fourier transform (FFT). Such techniques, although simple to implement, exhibit problems of FFT-based spectral analysis: inability to detect short (“phasic”) EEG events, unless the time window of the analysis is short as well (which may result in problems of frequency resolution), and difficulty in distinguishing between diffuse “background” activity in the spindle frequency band and well-defined spindles. These problems can be addressed by using time-frequency analysis techniques (e.g., wavelets) as well as matching pursuit procedures (which can be viewed as a generalization of wavelet analysis), although questions still remain as to the “best” mother wavelet or number and kind of atoms to use.

The above methods rely on the a priori knowledge of some electrographic characteristics defining the sleep spindle. The artificial neural network (ANN) approach for detection may not depend on any such explicit knowledge (e.g., Ventouras et al., 2005). However, the generalization capability of ANN-based methods, which cannot be evaluated analytically (as in an expert system-based method), is not “guaranteed” and it depends in a quite non-linear way on the structure of the ANN architecture and on the training data. Combinations of possibly more than one ANN systems as pre-processors allowing any “spindle-like” waveform to be further evaluated, followed by a knowledge-based system mimicking an expert (or a consensus of experts) for more elaborate analysis, appear to be promising approaches.

A successful detection system exhibits mostly true positive spindle detections (TPs) and very few false positive spindle detections (FPs). We define TP performance (TPP) as follows: (the number of TPs)/(the number of spindles detected by the visual scorers). If possible, the visually detected spindles should reflect the consensus of several scorers. Visual scoring is still the “gold standard” to compare automated detection systems to, despite the fact that experts often make mistakes, may be biased using ill-defined procedures, and may not be always consistent. We define FP performance (FPP) as follows: (the number of FPs)/(the sum of FPs and TPs). TPP and FPP figures should be provided for testing data, which should be separate from training data. Both training and testing data should contain records of several subjects, of various ages and pathologies, as sleep spindle morphology may vary as a function of age and pathology. In addition, the data should contain EEG epochs exhibiting various kinds of recording artifacts, such as movement and muscle (EMG) activity, since automated detection systems should be capable of analyzing routine sleep EEG records obtained in an artifact-prone clinical environment. Therefore, artifact detection or rejection capabilities should be incorporated into detection systems.

Deciding on “optimum” TPP and FPP figures is not straightforward. Satisfactory TPP and FPP figures should relate to the use of the automated system. For example, if the system is to be used for automated sleep staging in routine sleep EEG analysis, it may not be necessary to detect each and every sleep spindle, but enough of them so that a 30-s EEG epoch can be accurately assessed as sleep stage 2 (Rechtschaffen and Kales, 1968). However, in order not to misinterpret as sleep stage 2 other sleep stages where no spindles are expected, a “relatively good” FPP (say, less than 20%) may be appropriate. In cases where not missing spindles is of paramount importance, as in sleep EEG records of patients with neurological or psychiatric disorders where there is a paucity of spindles (e.g., dementia, schizophrenia), increasing TPP and decreasing FPP may be necessary. This could apply, for example, to clinical studies on the effect of pharmacotherapy in schizophrenia, where effects on thalamic centers involved in sleep spindle generation are investigated (Ferrarelli and Tononi, 2011). Assuming that high TPP and low FPP figures might necessitate a complicated system structure, it should be of interest to develop systems exhibiting some kind of modularity, whereby TPP and FPP could be altered depending on the use.

### Experimental and clinical studies

A reliable detection system can contribute to the effective and accurate quantification of sleep spindle occurrence patterns, either through spindle counts or spindle density figures (i.e., spindle number/time window of observation). It can also aid in topographical studies of “slow” and “fast” spindles, which should be of interest (Zeitlhofer et al., 1997), as well as contribute in tracking the propagation of sleep spindles across the scalp, for the study of sleep spindle dynamics (O'Reilly and Nielsen, 2014). In some cases, the spindle sequence pattern (e.g., how inter-spindle time intervals are distributed in time) might be of importance (Ktonas et al., 2000), especially if spindle generation mechanisms are being studied. There is evidence that sleep spindles are generated through the interaction of cortico-thalamo-cortical neuronal networks, and that the so-called Slow Wave Oscillation (SWO), a cortical EEG rhythm of frequency content less than 1 Hz, serves as a “pacemaker” for the thalamic reticular nucleus to generate spindles (Steriade and Amzica, 1998). Studying sequence patterns in inter-spindle time intervals can provide information about SWO intra-frequency dynamics which may relate to cortical processes of interest, such as learning and memory consolidation (Molle et al., 2011).

Systems should provide the capability of extracting specific electrographic parameters from the detected spindles, such as mean amplitude, intra-spindle frequency and spindle length, which may relate to EEG generating mechanisms possibly affected by an experimental procedure (e.g., sleep deprivation, pharmacotherapy) or a neurological/psychiatric disorder. Accordingly, any changes in spindle mean amplitude may relate to changes in cortical processes, while changes in intra-spindle frequency and spindle length may relate to changes in thalamic or thalamo-cortical processes (Steriade and Amzica, 1998). Given their electrographic shape, sleep spindles could be viewed as amplitude-modulated and frequency-modulated (AM/FM) signals. Therefore, methodology for the analysis of analytic signals (e.g., Hilbert transforms) as well as time-frequency analysis techniques provide the opportunity of extracting parameters related to the instantaneous envelope and instantaneous frequency of spindles, allowing the possibility to study pathological processes that might affect such parameters, as, for example, in schizophrenia, dementia and cognitive dysfunction (Ktonas et al., 2009; Ferrarelli and Tononi, 2011; Carvalho et al., 2014).

## Estimation of intracranial current sources for sleep spindles

### Estimation methods

The non-invasive estimation of intracranial current sources for sleep spindles can be achieved by solving the inverse bioelectromagnetic problem, based on scalp EEG or MEG (magnetoencephalography) measurements. The sources are usually modeled as current dipoles. In the equivalent current dipole (ECD) approach, the number, location, amplitude and orientation of dipoles are to be determined. A set of dipoles is selected which best conforms to an optimization criterion.

In the Distributed Source Model (DSM) approach no restrictions are imposed on the number of sources to be computed. Optimization techniques are adopted for solving this highly under-determined problem, incorporating mathematically and/or biophysically inspired restrictions, but without certainty that no distribution other than the selected one could be closer to the real underlying distribution. Low-Resolution Electromagnetic Tomography (LORETA) is a DSM method selecting the solution which minimizes the Laplacian of the depth-weighted sources. Based on the assumption that contiguous neuronal assemblies have correlated activity, LORETA provides solutions that might be “over-smoothed.” Since anatomically contiguous areas can be functionally distinct, concurrent activity in such contiguous areas must be dealt with attention when inspecting the results of LORETA. Other DSM methods, like dynamic SPM (dSPM) and standardized LORETA (sLORETA), compute statistical scores indicating locations where activity would occur with low error probability, therefore creating statistical parametric maps which can provide more focused loci of activity than LORETA. Taking into account the rather diffuse distribution of spindle cortical activity, DSM methods seem more appropriate for spindle source estimation than ECD methods, since ECD methods limit the number of sources that can be investigated and, in order to perform adequately, the number of sources must be inferred a priori (Michel et al., 2004).

### Experimental and clinical studies

Source estimation techniques can be used to elucidate plausible neural generation mechanisms for sleep spindles and, in particular, the electrogenesis of “slow” and “fast” spindles. LORETA based on EEG has provided indications that fast (slow) spindle source activity is located posteriorly (anteriorly) in the cortex (Durka et al., 2005; Ventouras et al., 2010). Studies based on MEG data (Manshanden et al., 2002; Urakami, 2008) have found that four source areas, located in parieto-central and fronto-central cortical regions, bilaterally, adequately explain most of the variation in spindles, although indications for considering both slow and fast spindle source activity as a single event were provided using MEG data (Gomenyuk et al., 2009). However, the inversion of simultaneous EEG and MEG recordings (Dehghani et al., 2010) has found that there are significant differences between sources derived from EEG and those derived from MEG.

Although there is some degree of similarity among the source areas detected by the various studies, there is a need for providing a comparative analysis of a comprehensive set of inversion methods applied to an extensive set of data because of the different principles on which the various methods operate. Along these lines, the concurrent recording of EEG and MEG should be pursued. Similarly, several studies have used concurrent EEG and fMRI recordings, investigating the fMRI-obtained brain activation during sleep spindles (Caporro et al., 2012). EEG/MEG modalities are generally restrained to cortical imaging. However, the generators of spindles are thought to be thalamic and, therefore, not accessible to EEG/MEG. Concurrent EEG and fMRI recordings can provide information on “spindle-coincident” activation in sub-cortical formations, such as the thalamus. Therefore, the limitations of the bioelectromagnetic inverse problem methodologies can be surpassed, providing indications for relations of “slow” and “fast” spindles to thalamic and cortical activity (Schabus et al., 2007). Consequently, such studies should be actively pursued and are expected to significantly elucidate spindle generation mechanisms.

Application of inversion techniques in patient populations should be encouraged, as in investigating the cortex involvement in the asymmetry of spindles after hemispheric stroke (Urakami, 2009) and the generation of spindles in temporal lobe epilepsy (Del Felice et al., 2013). A topic that has not yet been addressed concerns the extraction of parameters related to the phenomenology of intracranial current sources. Accordingly, it might be of interest to compute measures of current source spread and intensity as a function of time (along the duration of a spindle). Such approaches could help in differentiating healthy controls from patient populations, and in differentiating among various patient populations as well.

## Summary

This contribution provided comments on methodological issues related to the automated identification and characterization of sleep spindles and their intracranial sources, and to the understanding of their functional significance. Specific guidelines were presented for the computer-based detection and analysis of spindles and their intracranial sources, as well as for related experimental and clinical studies.

## Author contributions

Periklis Y. Ktonas and Errikos-Chaim Ventouras contributed to the conception and design of the work, as well as to drafting and critically revising it, and to providing final approval of the version to be published. They both agree to be accountable for all aspects of the work.

### Conflict of interest statement

The authors declare that the research was conducted in the absence of any commercial or financial relationships that could be construed as a potential conflict of interest.
